# The Passing of the Antitoxin

**Published:** 1895-07

**Authors:** 


### *EDITORIAL.*

---

The office of THE JOURNAL is in room 330 Equitable Building.

The editors are not responsible for views expressed by contributors.

Articles for publication should reach this office not later than the 15th instant.

Address all communications, and make all remittances payable to THE ATLANTA MEDICAL AND SURGICAL JOURNAL, Atlanta, Ga.

Reprints of articles will be furnished, when desired, at a nominal cost. Requests for the same should always be made *on the manuscript.*

---

### THE PASSING OF THE ANTITOXIN.

It has now been many months since the antitoxin-horse-blood-serum treatment of diphtheria was introduced and proclaimed to the profession amid wild enthusiasm; long enough certainly for those who formerly announced that the long-wished-for specific was at hand to have brought forward sufficient reason for the faith that was in them. Not since the memorable shout of Koch went up, some years ago, has such an *ex cathedra* proclamation been received with more eager attention and hopeful interest. The statements of Behring, Kossel, Ehrlich, Aronson, Wernicke, Roux, and other guinea-pig therapeutists, supported as they seemed to be by the invincible logic of statistics, were of the most assuring nature. It was even said by some that if the antitoxin treatment were begun early in the disease there need be no mortality any longer.

Indeed, we appeared to have arrived upon a new era in the treatment of this hitherto grave and formidable disease. But there were not long wanting those who opposed it unconditionally, as either negatively useless or positively injurious, and the conservative general practitioner was left at a loss to know what to believe, a state of being from which he has not yet completely recovered.

While the method has certainly failed in a large measure to fulfill the promises of its youth, and while late expressions of opinion as to its value have been tempered with much more moderation than formerly, it may yet prove to be a remedy of real value in certain cases. Variot says (*Journal de Clinique et de Therapeutique Infantile*, May 16) that the antitoxin may give brilliant results in ordinary cases of pharyngeal diphtheria, or in cases in which there is even laryngeal stenosis. Yet it fails in toxic cases or those suffering with systemic poisoning. While he disputes the antitoxic action of the serum, he admits that it antagonizes the false membranes, causing them to disappear. He thinks "we should not underrate the immense value of the antitoxin treatment." Baginsky and Katz have made an elaborate report of 167 cases lately treated and carefully studied. Their paper is strongly in favor of the treatment. Their opinion is that "while the antitoxin is not a cure-all, it will exert a most favorable influence in the majority of the worst cases of diphtheria." In the late work by Sternberg (*Immunity and Serum Therapy*) the author states that "Experimental and clinical evidence heretofore submitted appear to establish the value of the treatment when applied before the disease has progressed too far." Heubner, of Berlin, says that he can be "anything but skeptical as to the curative action of the serum in diphtheria." It is to be noted that those who advocate its use have ceased to regard it as the one specific capable alone of conquering the disease, but recommend it as a procedure which may be practiced in conjunction with the "usual other remedies."

On the other hand, it is said that in Germany, the home of the *heilserum*, there is now "almost no demand for the remedy in question." Hansemann, of Berlin, was the first to question the efficacy of the treatment, pointing out a large number of failures even where large doses of the serum had been used, also a number of relapses after apparent recovery, also the failure of the serum to protect some children against the disease who had been exposed to it, and also certain untoward effects of the serum upon the system.

The London *Medical Times* says that the treatment has been pronounced a failure in Vienna. The London correspondent of the *Medical News* says that there is a distinct lull in the excitement there over the antitoxin treatment, which he calls the "settling-down of turbid enthusiasm to the pure translucency of sober judgment."

Drascke has reported unfavorably upon the treatment, basing his opinion upon a study of thirty cases. He had not "met with a single instance in which he could feel convinced that the serum had produced any direct effect." In some cases the serum had affected the kidneys, and he had noted other symptoms which should warn medical men against its use. Dr. Winters, of New York, opposes it vigorously. He has not seen any evidence that a single symptom has been relieved, but he regards it as dangerous in its immediate effects on the kidneys and nerve centers and its remote effect on the blood. The *Medical Brief* says that it has not been conclusively shown in a single instance that recovery could be attributed to its use. Attention has been called to the facts that the treatment involves the introduction of a toxalbumin into the blood of a patient who may have already an excess of albumin in the blood, and that the introduction of the serum of one animal into the blood of another produces a dissolution of the red blood corpuscles of the latter. These facts have been offered as explanations of some of the unfavorable immediate effects of the injections.

Dr. William Jacobson, in a late letter to the *Medical Record* (May 11, 1895), has pointed out "How the Value of Antitoxin is Distrusted by Some of the Men Who Advocate its Use." He shows that these gentlemen have not been willing to discard the old remedies for the new, continuing to employ antiseptic sprays, tincture of iron, gray powder, turpeth mineral, digitalis, nux vomica, stimulants, and the other agents which have been our dependence in the past.

If called upon to actually define the true status of the remedy, perhaps we would be safe for the present in assuming a middle ground—that certain cases and certain symptoms seem to be benefited and relieved by the timely and judicious use of antitoxin, and that it may materially aid, but not displace, the usually-tried remedies. Its best friends can now hardly claim more than that; its opponents, or some of them, at least, will probably concede that much.

